# Identification and functional characterization of the chloride channel gene, *GsCLC-c2* from wild soybean

**DOI:** 10.1186/s12870-019-1732-z

**Published:** 2019-04-01

**Authors:** Peipei Wei, Benning Che, Like Shen, Yiqing Cui, Shengyan Wu, Cong Cheng, Feng Liu, Man-Wah Li, Bingjun Yu, Hon-Ming Lam

**Affiliations:** 1 Laboratory of Plant Stress Biology, College of Life Sciences, Nanjing Agricultural University, Nanjing, China; 2 State Key Laboratory of Crop Genetics and Germplasm Enhancement, College of Life Sciences, Nanjing Agricultural University, Nanjing, 210095 China; 3 Center for Soybean Research of the State Key Laboratory of Agrobiotechnology and School of Life Sciences, The Chinese University of Hong Kong, Shatin, Hong Kong, China

**Keywords:** Chloride, Chloride channels, CLCs, Differential expression, GsCLC-c2, Nitrate, Salt stress, Soybean, Wild soybean

## Abstract

**Background:**

The anionic toxicity of plants under salt stress is mainly caused by chloride (Cl^−^). Thus Cl^−^ influx, transport and their regulatory mechanisms should be one of the most important aspects of plant salt tolerance studies, but are often sidelined by the focus on sodium (Na^+^) toxicity and its associated adaptations. Plant chloride channels (CLCs) are transport proteins for anions including Cl^−^ and nitrate (NO_3_^−^), and are critical for nutrition uptake and transport, adjustment of cellular turgor, stomatal movement, signal transduction, and Cl^−^ and NO_3_^−^ homeostasis under salt stress.

**Results:**

Among the eight soybean *CLC* genes, the tonoplast-localized *c2* has uniquely different transcriptional patterns between cultivated soybean N23674 and wild soybean BB52. Using soybean hairy root transformation, we found that *GsCLC-c2* over-expression contributed to Cl^−^ and NO_3_^−^ homeostasis, and therefore conferred salt tolerance, through increasing the accumulation of Cl^−^ in the roots, thereby reducing their transportation to the shoots where most of the cellular damages occur. Also, by keeping relatively high levels of NO_3_^−^ in the aerial part of the plant, *GsCLC-c2* could reduce the Cl^−^/NO_3_^−^ ratio. Wild type GsCLC-c2, but not its mutants (*S184P*, *E227V* and *E294G*) with mutations in the conserved domains, is able to complement *Saccharomyces cerevisiae △gef1* Cl^−^ sensitive phenotype. Using two-electrode voltage clamp on *Xenopus laevis* oocytes injected with *GsCLC-c2* cRNA, we found that GsCLC-c2 transports both Cl^−^ and NO_3_^−^ with slightly different affinity, and the affinity toward Cl^−^ was pH-independent.

**Conclusion:**

This study revealed that the expression of *GsCLC-c2* is induced by NaCl-stress in the root of wild soybean. The tonoplast localized GsCLC-c2 transports Cl^−^ with a higher affinity than NO_3_^−^ in a pH-independent fashion. GsCLC-c2 probably alleviates salt stress *in planta* through the sequestration of excess Cl^−^ into the vacuoles of root cells and thus preventing Cl^−^ from entering the shoots where it could result in cellular damages.

**Electronic supplementary material:**

The online version of this article (10.1186/s12870-019-1732-z) contains supplementary material, which is available to authorized users.

## Background

Salinization of irrigation water and soil is one of the most serious environmental factors limiting crop productivity worldwide [1–3]. Sodium chloride (NaCl) is the most common salt found in saline soil. It hinders plant growth not only through ionic toxicity but also by causing water deficit inside the plant, i.e. osmotic stress, and imbalance or deficiency of other ions such as potassium (K^+^) and nitrate (NO_3_^−^) [4–6]. Plants developed different strategies to combat salt stress, including exclusion or compartmentalization of ions to reduce cytosolic Na^+^ and Cl^−^, especially in the aerial parts [7, 8]. Generally, crops such as cotton, rice and barley are more sensitive to Na^+^ than Cl^−^, whereas tobacco, grape, potato, citrus and cultivated soybean, in which salt stress effects are mainly caused by Cl^−^, are known as “Cl^−^ -sensitive” or “Cl^−^ -hating” plants [8–15]. So far, researches on the physiological and molecular mechanisms of salt tolerance in plants have mostly focused on Na^+^ toxicity and adaptations. However, until recently, the salt injury caused by Cl^−^ has been largely ignored [11, 14].

Cl^−^, as one of the essential micronutrient elements, is one of the main anions in plant cells besides NO_3_^−^, and is normally accumulated to macronutrient levels for enhancing plant growth and development. Cl^−^ is involved in photosynthesis by stabilizing the water splitting system or oxygen-evolving complex of photosystem II (PSII), and also in stomatal movement, cellular osmotic pressure maintenance, electrical charge balance, turgor and pH regulation, nitrogen use efficiency, water-holding capacity and disease resistance [5, 14–16]. Chloride deficiency leads to reduced plant growth and symptoms such as leaf chlorosis [15]. Despite its beneficial roles in plant nutrition, Cl^−^ can be a major toxic element in the cytosol when plants are grown in salt-affected soils, where Cl^−^ is predominant and can accumulate in excess in the shoot/leaf, adversely affecting plant growth [6, 15]. During salt stress, the effects of Cl^−^ can be additive or synergistic to those of Na^+^ [11]. Excessive amounts of Cl^−^ can lead to decreased uptake of NO_3_^−^, which is the most important nitrogen source for plants and the main monovalent anion in plant tissues and cells next to Cl^−^ [14, 17, 18]. Both NO_3_^−^ and Cl^−^ are monovalent anions with similar ionic radii, and perform a similar role in maintaining cellular charge balance and turgor, and can often be transported by the same proteins [11]. To combat Cl^−^/salt stress in plants, an increased NO_3_^−^:Cl^−^ ratio in the tissues or organs, similar to the well-described increased K^+^:Na^+^ ratio in shoots, can play a positive role [9, 14, 19, 20]. Although chloride is a beneficial micronutrient in its own right and not simply a “cheap osmoticum” for plants [15], its impacts on plants under salt stress are problematic [11]. The nature of Cl^−^ toxicity or the mechanisms of Cl^−^ transport and detoxification during salt stress is far from being understood to such degrees as those of Na^+^ [14, 21]. Despite the general lack of information, a few reports have suggested that the control of Cl^−^ transport from roots to shoots or the ability to maintain a low Cl^−^ level in shoots is the key determinant of Cl^−^/salt tolerance in plants [8, 10, 12]. In the past few years, effects of Cl^−^ toxicity and functions of some essential Cl^−^ transporters, such as the chloride channel proteins (CLCs), that mediate Cl^−^ transport and homeostasis in plants under salt stress are drawing increasing research interests [11, 15].

Currently, *CLC* genes have been identified in the genome of higher plants including *Arabidopsis*, tobacco, rice, potato, citrus, soybean, maize, and poplar with 7–8 gene members in each species (summarized in [21, 22]). In total, 217 homologous CLC protein or gene sequences belonging to 34 species of plants have been retrieved from the Pfam database (http://pfam.xfam.org/), Uniprot database (http://www.uniprot.org/), and NCBI database [22, 23]. The large number of plant CLC proteins, with their dual functions of NO_3_^−^ and Cl^−^ transport, and the differences in their respective affinity for NO_3_^−^ or Cl^−^ pose a degree of technical difficulties and uncertainties in the results from in-depth researches [24–26].

The genetic diversity between cultivated crops and their wild relatives provides rich resources for trait and gene discovery that have yet to be sufficiently utilized. The cultivated soybean (*G. max*) is the most important legume crop in the world, offering high-quality protein and oil for human food and animal feed [27]. When exposed to NaCl stress, Cl^−^ in soybean plants is usually accumulated to a toxic level in the shoots ahead of Na^+^. Our previous work suggested that although both Na^+^ and Cl^−^ are toxic to soybean under salt stress, there are differences between the sensitivity to Na^+^ and Cl^−^ for *G. max* and *G. soja* (the wild soybean) [8, 12, 28]. Cl^−^ toxicity is more deleterious than Na^+^ to the cultivated soybean and the damage is positively related to the Cl^−^ contents within the leaves and stems, whereas the wild soybean and its hybrids with the cultivated soybean have stronger Cl^−^ tolerance than the cultivated soybean itself [8, 12, 28].

*GmCLC1* (GenBank accession: AY972079; Phytozome database: Glyma.05G077100) encodes a tonoplast-localized and pH-dependent Cl^−^/H^+^ antiporter, which was upregulated by NaCl or dehydration stress, and *GmCLC1*-transgenic tobacco BY-2 cells displayed enhanced NaCl tolerance through increased Cl^−^ transport from the cytoplasm into the vacuole [29, 30]. Our prior study also found that *GmCLC1-*transgenic *Arabidopsis thaliana* and *GmCLC1-*overexpressing soybean hairy roots had enhanced Cl^−^/salt tolerance by reducing the Cl^−^ accumulation in shoots or by sequestering more Cl^−^ in roots [21]. The survival rate of *GmCLC1*-transgenic *Δgef1* mutant yeast cells was also increased under varied levels of Cl^−^/salt stress [21]. Based on published *GmCLC1* information and public databases, we found seven other homologous *CLC* members, *b1*, *b2, c1, c2, d1, d2 and g*, in the soybean genome in both wild and cultivated soybean [22].

In this work, all the homologous soybean *CLC* members were cloned from the Cl^−^-sensitive *G. max* cultivar N23674 and the Cl^−^-tolerant *G. soja* accession BB52. By examining their expressions under favorable or NaCl-treated conditions, we isolated *GsCLC-c2*, which is the only CLC member having non-synonymous changes between *G. max* N23674 and *G. soja* BB52. We have also systemically investigated its functions using hairy root-composite soybean plants, site-directed mutagenesis (SDM)-mediated yeast mutant complementation, and electrophysiological assays with *Xenopus laevis* oocytes. Our goal is to decipher the molecular and physiological roles of *GsCLC-c2* in soybean Cl^−^/salt stress adaptations, so as to provide an important theoretic basis for improving Cl^−^/salt tolerance in soybean and other crops.

## Results

### Whole-genome analyses of *CLC* homologous genes in *G. max* and *G. soja*

**Fig. 1:**
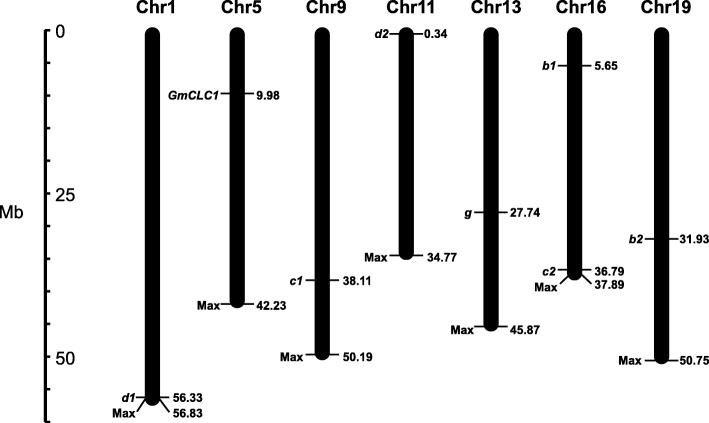
Genome-wide chromosomal locations of soybean *CLC*-homologous genes. Number on the right of the chromosome indicate the position of the gene in megabases (Mb). Max: total length of the chromosome

Using the Phytozome database (https://phytozome.jgi.doe.gov/pz/portal.html), we have previously found seven homologs of *GmCLC* (Glyma.16G057600, Glyma.19G089800, Glyma.09G157900, Glyma.16G208400, Glyma.01G239000, Glyma.11G004600 and Glyma.13G161800) in the soybean genomes of both wild and cultivated soybeans, and named them *b1*, *b2*, *c1*, *c2*, *d1*, *d2* and *g* [22]. Here we extracted total RNA from the roots, stems and leaves of *G. max* cultivar N23674 and *G. soja* accession BB52 15-day-old seedlings, produced cDNAs by reverse transcription of mRNAs, and successfully cloned these seven new *CLC* homologs. These eight soybean *CLC* homologs (including *GmCLC1*) are located on chromosomes 1, 5, 9, 11, 13, 16 and 19, with two of the members, *b1* and *c2*, both being on chromosome 16 (Fig. 1). The coding sequence (CDS) lengths of these *CLCs* are between 2178 and 2472 bases. With the exceptions of *GmCLC1*, *c1*, and *d1* (which are identical between the wild and cultivated soybeans), a few synonymous nucleotide substitutions were found in the other *CLC* members between *G. max* cultivar N23674 and *G. soja* accession. The only exception is *c2* where there is a 462 T > A substitution from cultivar N23674 to accession BB52, which resulted in the corresponding D154E change (Additional file 1: Table S1).

### Differential expressions of *CLC*s in *G. max* and *G. soja* under NaCl stress

**Fig. 2:**
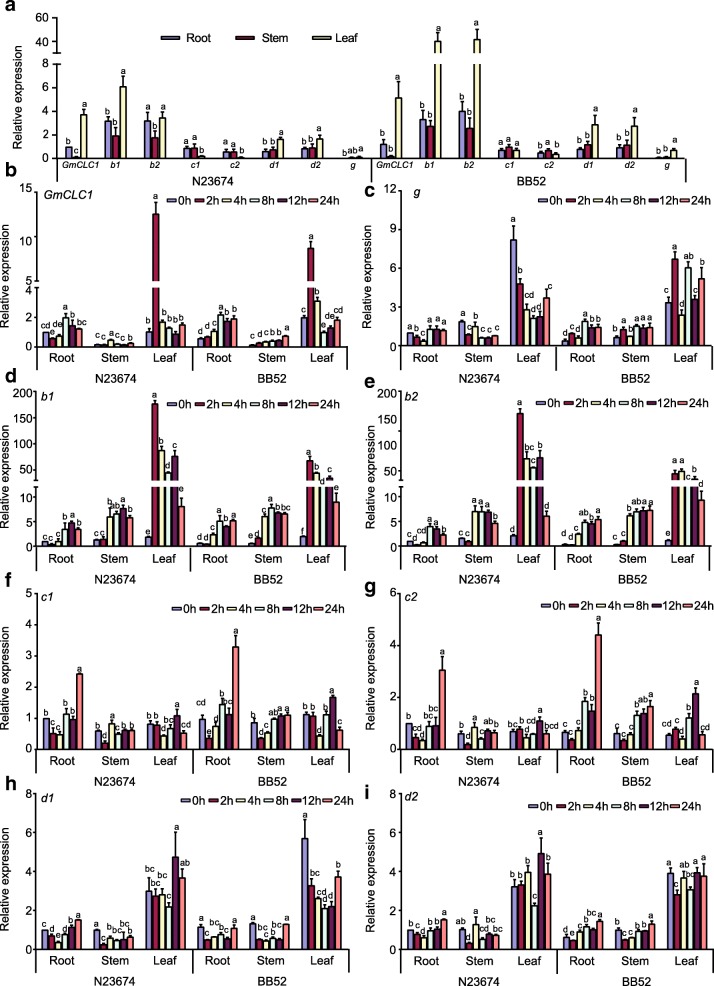
Relative expressions of *CLC-*homologous genes in *G. max* N23674 and *G. soja* BB52 15-day-old seedlings. **a** Under normal conditions. All expressions were normalized to the expression of *GmCLC1* in root of N23674. Expression of *GmCLC1* in root in N23674 was set as 1 for comparison. **b**-**i** Expression of different *CLC*-homologs under 150 mM NaCl stress for 0, 2, 4, 8, 12, and 24 h. *g, b1, b2, c1, c2, d1* and *d2* are *CLC* homologs. The transcript levels have been normalized against those of *GmEF1α2a*. Expression of the gene in root of N23674 was set as 1 for comparison. The results were presented as mean ± SD (3 ≤ *n* ≤ 5). Letters indicate groups with statistically significant differences (*P* ≤ 0.05) using Duncan’s test after one-way ANOVA

Under normal growth conditions, all eight *CLC* homologs were expressed in roots, stems and leaves of 15-day-old seedlings of *G. max* cultivar N23674 and *G. soja* accession BB52. Generally, the relative expressions in the leaves of most of the *CLC* homologs, including *GmCLC1* [21, 29, 30], *b1*, *b2*, *d1*, *d2* and *g*, were markedly higher than those in the roots and stems of both wild and cultivated soybeans. However, the relative expressions of *c1* and *c2* showed a different trend. Expression of both *c1* and *c2* was lower in leaf than in root and stem of N23674 (Fig. 2a). Expression of *c1* in BB52 showed no significant difference in leaf, stem and root. But expression of *c2* was significantly higher in stem compared to that in root and leaf (Fig. 2a). When the plants were treated with 150 mM NaCl, *GmCLC1*, *b1* and *b2* were initially up-regulated (for the first 2 h) and were then down-regulated quickly in the leaves of both N23674 and BB52, whereas the expression of member *g* in the leaves declined gradually with increasing salt treatment time in N23674 but fluctuated in the leaves of BB52 (Fig. 2b-e). The expressions of *GmCLC1*, *b1* and *b2* were slightly up-regulated in the roots and stems of both wild and cultivated soybean seedlings under salt stress after 8 h (Fig. 2b-e). With respect to *d1* and *d2*, their expressions decreased after the first 2 h in the roots and stems and after 8 h in the leaves, and then increased again with longer salt treatment time to about the same level as before the salt treatment (Fig. 2h-i). On the other hand, the expressions of *c1* and *c2* in the leaves peaked after 12 h of NaCl exposure and then dropped back to the level before salt treatment (Fig. 2f-g). More significantly, their expressions in the roots increased more than 2-fold after 24 h of salt treatment, in both N23674 and BB52 (Fig. 2f-g). The distinctively different transcriptional responses of *c1* and *c2*, in the different tissues under both normal and salt-stress conditions and in both wild and cultivated soybeans, from those of the other soybean *CLC* members make them warrant further investigation. Between the two, *c2* is the only one with a difference in amino acid sequences between *G. max* cultivar N23674 and *G. soja* accession BB52 (Additional file 1: Table S1). Therefore, we decided to examine in greater detail what physiological (including electrophysiological) and molecular roles the version of *c2* found in *G. soja* BB52 (heretofore to be named *GsCLC-c2*) might play in soybean Cl^−^/salt tolerance.

### Certain site-directed mutagenesis (SDM) mutants of *GsCLC-c2* failed to complement the chloride channel-deficient yeast mutant *Δgef1* under NaCl, KCl, or KNO_3_ treatment

**Fig. 3:**
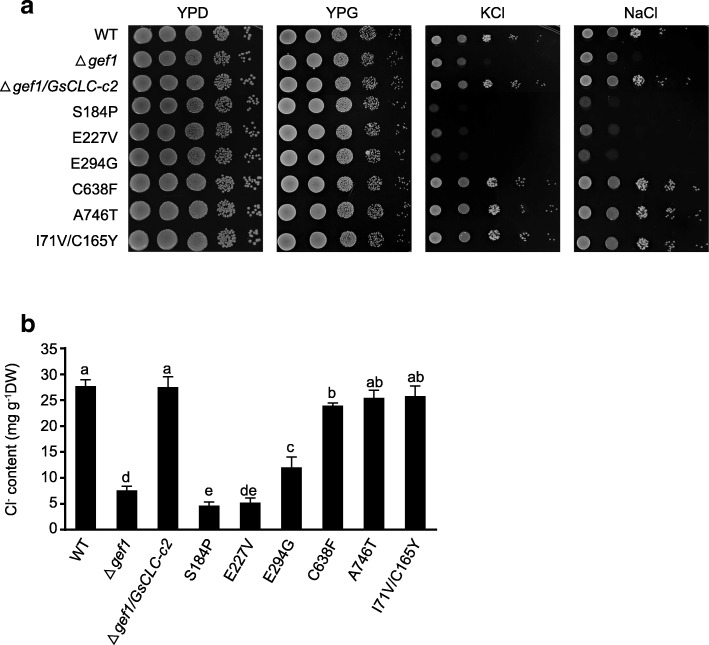
Effects of expressing wild type or mutant *GsCLC-c2* on salt-sensitive yeast mutant *Δgef1* under salt treatments. **a** Survival in YPD and YPG media, and YPG media supplemented with 1 M KCl or 1 M NaCl. **b** Cl^−^ contents in yeast cells under 1 M NaCl treatment. S184P, E227V, E294G, C638F, A746T and I71V/C165Y are mutants with specific amino acid substituted. YPD:1% yeast extract/2% peptone/2% dextrose; YPG:1% yeast extract/2% peptone/2% galactose. Each bar represents mean ± SD of ion contents of three independent cultures of each strain. Letters indicate groups with statistically significant differences (*P* ≤ 0.05) using Duncan’s test after one-way ANOVA

All the yeast strains, including the WT (BY4741), *Δgef1* (a GEF1-deficient, salt-sensitive mutant), and *Δgef1* transformed with *GsCLC-c2* or SDM mutants of *GsCLC-c2* (*S184P*, *E227V*, *E294G*, *C638F*, *A746T* and *I71V/C165Y*), grew well on both YPD (yeast extract, peptone, dextrose) and YPG (yeast extract, peptone, galactose) media under favorable conditions. When cultured on YPG medium supplemented with 1 M KCl, or NaCl, the *Δgef1* mutant was unable to grow, but this phenotype was rescued by transformation with wild type *GsCLC-c2*. However, mutants transformed with *S184P*, *E227V*, and *E294G*, with the key residues of the conserved domains I, II and III, respectively, of *GsCLC-c2* mutated, failed to complement the growth defect of yeast cells in the presence of high concentrations of chloride salts. This suggested that these mutants could not mediate Cl^−^ homeostasis in yeast, and therefore these specific residues may be important for the anion transport activity of GsCLC-c2. The other *GsCLC-c2* mutants, *C638F*, *A746T*, and *I71V/C165Y*, however, could complement the *Δgef1* mutation, similar to the *Δgef1* mutants transformed with the wild type *GsCLC-c2* (Fig. 3a). The Cl^−^ contents of WT, *Δgef1*/*GsCLC-c2* and *Δgef1*/*GsCLC-c2* mutants (*C638F*, *A746T*, and *I71V/C165Y*) grown in 1 M NaCl were significantly higher than those of the *Δgefl* mutants or *Δgef* mutants transformed with *S184P*, *E227V*, and *E294G* (*P* < 0.05), and the *Δgef* mutants transformed with *S184P* or *E227V* had similar Cl^−^ levels as the untransformed *Δgef1* mutants (Fig. 3b). This indicates that GsCLC-c2 has a similar function to the yeast GEF1 in Cl^−^ transport, and that the S184P and E227V substitutions completely, and the E294G substitution partially, negated the Cl^−^ transport function of GsCLC-c2.

### *GsCLC-c2* alleviates salt injury on transformed soybean hairy root composite plants by regulating the contents of Na^+^, K^+^, Cl^−^, NO_3_^−^ and ratios of Cl^−^/NO_3_^−^ and Na^+^/K^+^

**Fig. 4:**
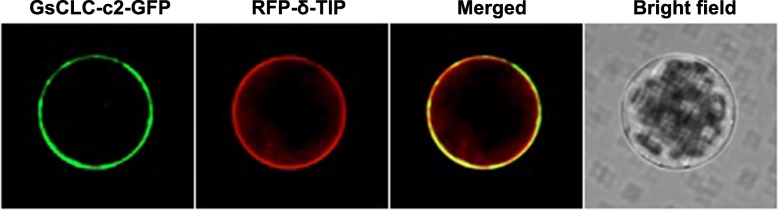
Subcellular co-localization of GsCLC-c2-GFP and δ-TIP-RFP recombinant proteins in leaf protoplasts of *Arabidopsis* seedlings. *GsCLC-c2-GFP* and *δ-TIP-RFP* were expressed in *Arabidopsis* leaf protoplasts under the control of CaMV-35S promoter. δ-TIP-RFP was used as the tonoplast marker

Using confocal microscopy, majority of GFP tagged GsCLC-c2 was found co-localized with the RFP tagged tonoplast marker protein δ-TIP (Fig. 4) suggesting that GsCLC-c2 may serve similar functions as other tonoplast localized CLCs.

**Fig. 5:**
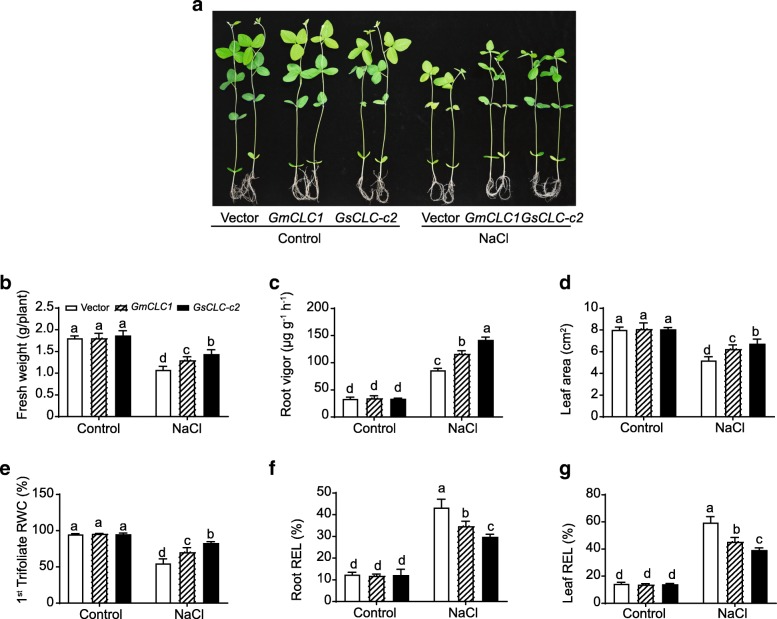
Comparisons of salt stress-related physiological parameters in *GsCLC-c2*- and *GmCLC1*-transgenic soybean hairy root-composite seedlings. **a** Photo showing the hairy root-composite seedlings with or without NaCl treatment. **b** Fresh weight, (**c**) root vigor, (**d**) leaf area, (**e**) relative water content (RWC) of the 1st trifoliate, (**f**) root relative electrolyte leakage (REL), and (**g**) leaf REL were compared between vector-transformed-, *GsCLC-c2*- and *GmCLC1*-transgenic soybean hairy root-composite seedling with or without NaCl treatment. Results are presented as mean ± SD (*n* = 3). Data of different lines under control and NaCl treatment were analyzed together by one-way ANOVA followed by Duncan’s test. Letters indicate groups with statistically significant differences *P* ≤ 0.05

To confirm the function of GsCLC-c2, we adopted the soybean hairy root-composite plant system. Composite plants expressing *GmCLC1* were used for comparison. For each parameter, multiple comparisons were performed to compare the performance of all transgenic lines under control and treatment conditions using one-way ANOVA. Under favorable (control) conditions, the empty vector-transformed, *GsCLC-c2-*transformed and *GmCLC1*-transformed hairy root soybean (*G. max* cv. N23674) composite plants all grew well with no observable phenotypic difference in term of fresh weight, root vigor, leaf area, relative water content (RWC) in first trifoliate, and relative electrolyte leakage (REL) in root and leaf (Fig. 5). When subjected to 120 mM NaCl solution for 7 d, all parameters were significantly affected compared to the control condition regardless of the transgene. The *GsCLC-c2-*transformed plants showed significantly better performance in fresh weight, root vigor, leaf area and RWC in the first trifoliate compared with the vector only control and the *GmCLC1*-transformed plants (Fig. 5b-e). Plant transformed with *GsCLC-c2* also showed less REL suggesting that they were under less salt damage at cellular level in contrast to the vector only control and the *GmCLC1-*transformed plant (Fig. 5f-g).

**Fig. 6:**
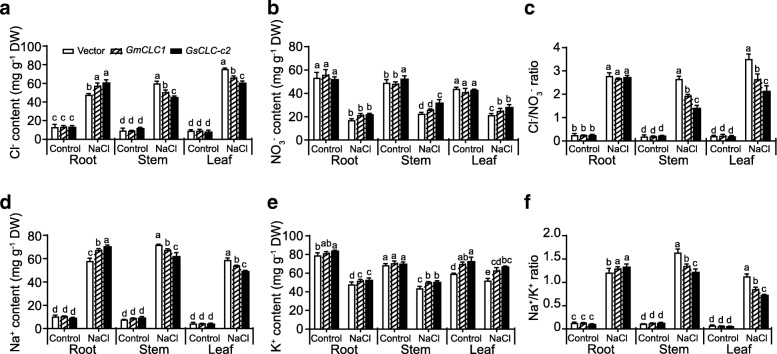
Ion content in different tissues of transgenic hairy root-composite plant. **a** Cl^−^ content, (**b**) NO_3_^−^ content, (**c**) Cl^−^/NO_3_^−^ ratio, (**d**) Na^+^ content, (**e**) K^+^ content, and (**f**) Na^+^/K^+^ ratio of transgenic hairy root-composite plants. Composite plants with hairy root transformed with vector, *GmCLC1*, and *GsCLC-c2* were employed in this study. Results are presented as mean ± SD (*n* = 3). Data of different lines within the same tissue under control and NaCl treatment were analyzed together by one-way ANOVA followed by Duncan’s test. Letters indicate groups with statistically significant differences *P* ≤ 0.05

To confirm the salt tolerance was brought about by the primary functions of *GsCLC-c2*, we also investigated the ion content in the transgenic composite plants. For each parameter, multiple comparisons were made to compare the performance of all transgenic lines in each tissue under control and treatment conditions using one-way ANOVA. Under control conditions, there was no significant difference in Cl^−^, Na^+^, Cl^−^/NO_3_^−^ and Na^+^/K^+^ ratio between the three constructs. Under the same conditions, composite plant of *GsCLC-c2* showed significantly higher NO_3_^−^ content in stem and K^+^ content in root and leaf. Under salt stress, all lines showed elevation of Cl^−^ and Na^+^ contents but reduction in NO_3_^−^ and K^+^ contents. Both *CLC*s expressing plants showed higher Cl^−^ content (accompanying with a higher Na^+^ content) in the root but a reduction of Cl^−^ content (accompanying with a reduction of Na^+^ content) in the aerial parts (Fig. 6a and d). With respect to NO_3_^−^, its level is higher in stem and leaf of composite plant ectopically expressing *GsCLC-c2* under salt stress conditions (Fig. 6b). Accordingly, ectopic expression of *GsCLC-c2* maintained significantly lower Cl^−^/NO_3_^−^ and Na^+^/K^+^ ratios in stem and leaf (Fig. 6c-f). Again, like the physiological parameters, *GsCLC-c2* transgenic plants accumulated a significantly lower Cl^−^ content in the aerial parts compared with the vector-transformed and *GmCLC1*-transformed plant (Fig. 6a) suggested that GsCLC-c2, under salt stress, has stronger ion transportation activities over GmCLC1.

### Electrophysiological analyses of *Xenopus laevis* oocytes expressing GsCLC-c2

**Fig. 7:**
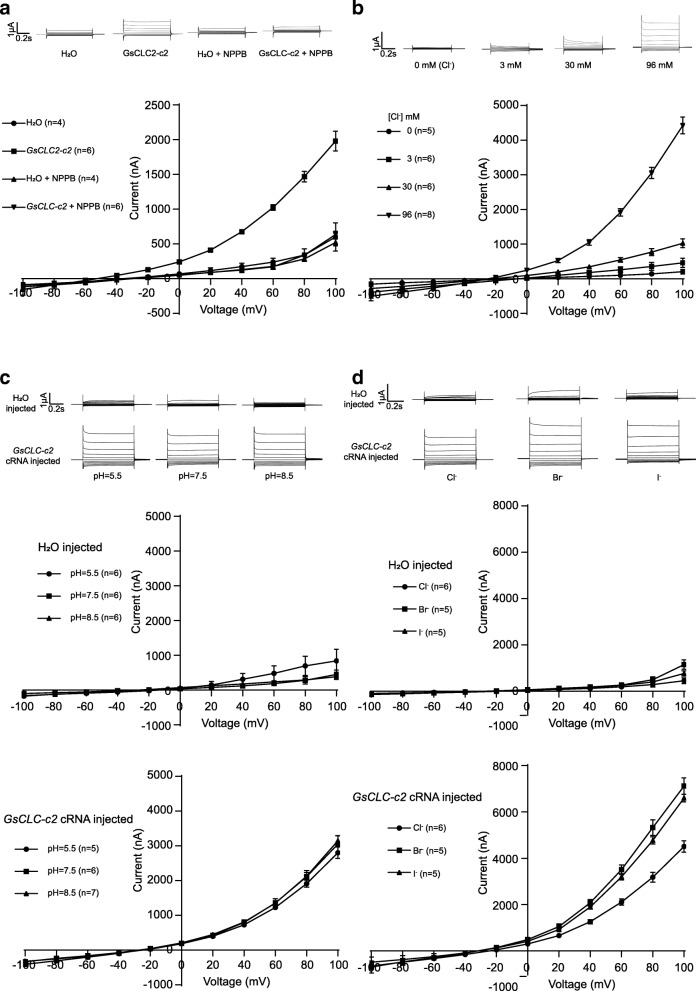
Voltage clamp analyses of *GsCLC-c2* expressions in *Xenopus laevis* oocytes. **a** Effects of NPPB. Water- or *GsCLC2-c2-* cRNA injected oocytes were incubated in standard bath solution (96 mM NaCl) with or without the anion channel blocker NPPB. **b** Effects of Cl^−^ concentrations. *GsCLC2-c2* cRNA injected oocytes were incubated in bath solution containing 0, 3, 30 or 96 mM NaCl. **c** pH effect. *GsCLC2-c2* cRNA injected oocytes were incubated in standard bath solution containing 96 mM NaCl at pH 5.5, 7.5, or 8.5. **d** Halide ion selectivity. Cells were kept in a bath solution containing 96 mM NaCl, NaBr or NaI. Results are presented as mean ± SD (*n* ≥ 4)

In order to further verify the anion transport activities of GsCLC-c2 to see if it is a bona fide anion channel protein, two-electrode voltage clamp experiments were performed using *Xenopus laevis* oocytes injected with *GsCLC-c2* cRNAs, and the electrophysiological track records for membrane steady-state current changes were conducted in vivo at 20 mV intervals between − 100 mV and + 100 mV. Oocytes microinjected with water (H_2_O) were used as the negative control to monitor the basal current. The results showed that, with the increase in voltage from − 100 mV to + 100 mV, the current increased at a much higher rate in the *GsCLC-c2*-transformed oocytes than the water-injected controls, reflecting a significant increase in the rate of change in conductance across the cell membrane. The invert rectifying current signified the movement of anion into or cation out of the oocytes. When the anion channel inhibitor 5-nitro-2-(3-phenylpropylamino) benzoic acid (NPPB) was added to the extracellular bath solution, the voltage-dependent change in current was not significantly different from the negative controls (H_2_O and H_2_O + NPPB) (Fig. 7a). Therefore, this demonstrates that GsCLC-c2 possesses anion transport activity, and that anion is very likely Cl^−^, since NPPB is a well-known chloride channel blocker [31].

To confirm the voltage-dependent currents induced by GsCLC-c2 was mainly due to its Cl^−^ transport activity, the currents across the membranes of *GsCLC-c2*-transformed oocytes, kept in Cl^−^ solutions at different concentrations (0, 3, 30, 96 mM), were recorded. The rate of increase in membrane currents was in direct proportion to the increase in ambient Cl^−^ concentration (Fig. 7b, Additional file 1: Figure S1), indicating that the anion transport activity of GsCLC-c2 was positively correlated with Cl^−^ concentrations. However, this activity was independent of pH at the levels tested (pH 5.5, 7.5 and 8.5) (Fig. 7c). This indicates that the Cl^−^ transport activity of GsCLC-c2 may not be coupled with H^+^ exchange. In addition, to investigate the affinity of GsCLC-c2 for different halide ions, *GsCLC-c2*-transformed oocytes were subjected to the standard bath solutions containing Br^−^, I^−^ or Cl^−^ at the same concentration. A voltage-dependent conductance response curve was obtained for all three ions, and the conductance responses to Br^−^ and I^−^ were about the same, and both were greater than that to Cl^−^ (Fig. 7d).

**Fig. 8:**
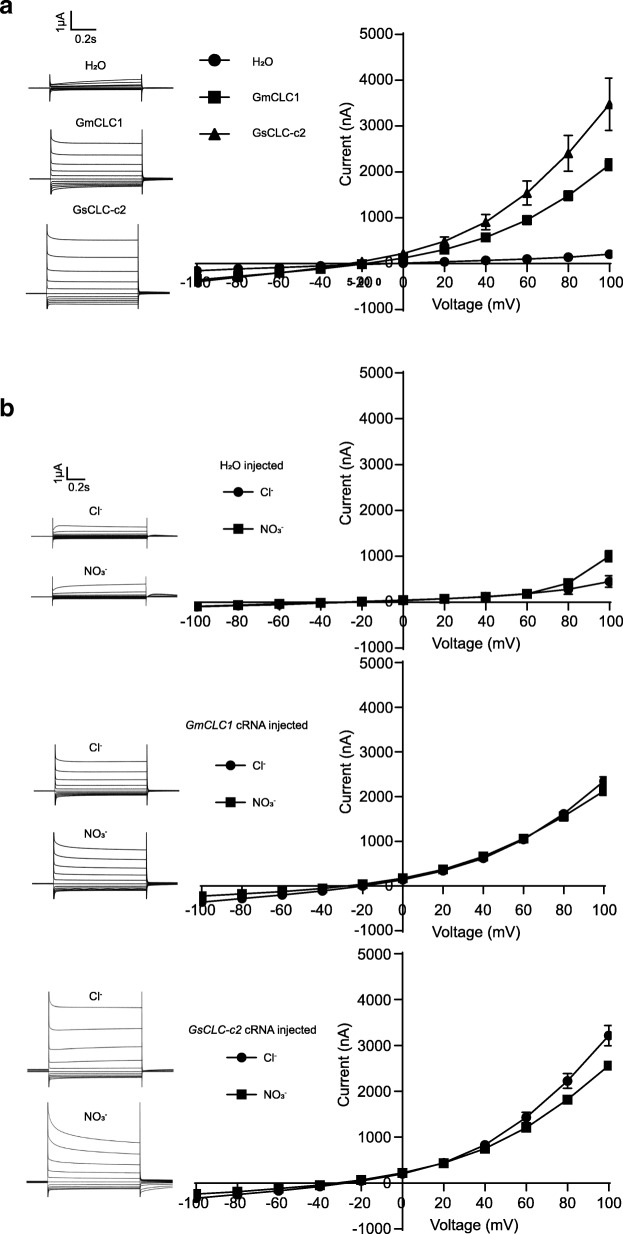
Electrophysiological comparisons of *Xenopus laevis* oocytes expressing *GsCLC-c2* and *GmCLC1*. **a** Ion transport activity in a bath solution containing 96 mM NaCl. **b** Cl^−^ and NO_3_^−^ selectivity in a bath solution containing 96 mM NaCl or and 96 mM NaNO_3_, respectively. Results are presented as mean ± SD (*n* ≥ 5). Upper panel: oocytes were injected with water; middle panel: oocytes were injected with *GmCLC1* cRNA; lower panel: oocytes were injected with *GsCLC-c2* cRNA

Because of the discrepancy between the anion transport activity of GsCLC-c2 reported here and that of the previously reported GmCLC1 with respect to pH dependence, we decided to directly compare the Cl^−^ transport activity between GsCLC-c2 and GmCLC1. Both *GsCLC-c2*-transformed as well as *GmCLC1*-transformed oocytes responded to the extracellular Cl^−^ bath solutions with significantly higher rates of change in conductance with increasing voltage compared to water control. However, the increase in currents induced by GsCLC-c2 was significantly steeper than that by GmCLC1, which demonstrates that the Cl^−^ transport activity of GsCLC-c2 is higher than that of GmCLC1 (Fig. 8a, Additional file 1: Figure S2). When *GsCLC-c2*-cRNA-transformed as well as *GmCLC1*-cRNA-transformed oocytes were exposed to either Cl^−^ or NO_3_^−^ bath solutions with the same anion concentrations, *GmCLC1*-cRNA-transformed oocytes did not show any significant difference between the current response curves to Cl^−^ and to NO_3_^−^, but the response curve for Cl^−^ was significantly steeper than that for NO_3_^−^ in the *GsCLC-c2*-transformed oocytes. The signal also attenuated faster for NO_3_^−^ than for Cl^−^ with GsCLC-c2 (Fig. 8b). This indicates that, different from GmCLC1, GsCLC-c2 shows a slightly higher affinity for Cl^−^ than for NO_3_^−^.

## Discussion

Investigations into Cl^−^ toxicity have been relatively sparse compared to those on the effects of Na^+^ on plants under salt stress [11, 14, 20, 32]. One of the few examples is the discovery that the rootstocks from salt-tolerant species of citrus and grape played a critical role in enhancing the Cl^−^/salt tolerance of the “chloride-sensitive” or “chloride-hating” seedlings [33–35]. Other studies have found that the key steps for alleviating Cl^−^ toxicity involve the sequestering of Cl^−^ in the root xylem parenchyma cells to limit its transport from the roots to the aerial parts, and the efflux of Cl^−^ by the root stele cells or compartmentalization of Cl^−^ in the vacuoles to reduce its availability in the roots [11, 36]. CLCs can play important roles in these processes and contribute to Cl^−^ homeostasis and Cl^−^/salt tolerance of plants [24, 32, 37, 38]. In Arabidopsis, there are seven members in the CLC protein family but only AtCLCc and AtCLCg (with 62% amino acid sequence identity to each other) have been shown to participate in Cl^−^ homeostasis, stomatal movement and Cl^−^/salt tolerance [24, 32, 37, 39]. The ectopic expression of *CsCLCc* from trifoliate orange (*Citrus trifoliata*) in *Atclc-c* mutant plants displayed improved seed germination, better growth and lower Cl^−^ accumulation in the roots and shoots than the non-transgenic mutant seedlings under salt stress [38].

In this study, the other seven members besides *GmCLC1* (*b1*, *b2, c1, c2, d1, d2* and *g*) of the soybean *CLC* gene family found in both *G. max* (cultivar N23674) and *G. soja* (accession BB52) have been successfully cloned for the first time and their sequences verified (Fig. 1, Additional file 1: Table S1). Specifically, *c2* is the only one that has an amino acid change between the cultivated (cultivar N23674) and the wild soybean (accession BB52): D154E, through a single nucleotide substitution (c.462 T > A) (Additional file 1: Table S1). At present, it is not clear whether this specific variation alone is responsible for the Cl^−^/salt tolerance differences between the cultivated and wild soybeans. However, based on the unique patterns of expressions of *c1* and *c2* in the roots, stems and leaves of N23674 and BB52 seedlings under both favorable and salt-stress conditions (Fig. 2), we postulate that *GsCLC-c2* may be more responsible for soybean responses to Cl^−^/NaCl stress in root than other *CLC* members. Similar to GmCLC1, GsCLC-c2 is also localized to the tonoplast (Fig. 4). Under NaCl stress, the salt damage to *GsCLC-c2*-overexpressing hairy roots composite soybean plants like the *GmCLC1*-overexpressing ones was clearly alleviated when compared to the empty vector-transformed plants (Fig. 5a). If we just look at the ionic effects of salt injury, this alleviation was correlated with the significant reduction in Cl^−^ contents of the stems and leaves of salt-stressed soybean plants, and indirectly related to the maintenance of NO_3_^−^ accumulation in various parts of the plants [9, 21, 22]. These combined phenomena can effectively reduce the Cl^−^/NO_3_^−^ ratios in the stems and leaves of the plants (Fig. 6c). Based on the results from the *Δgef1* yeast mutant assays (Fig. 3) and its localization on the tonoplast of *Arabidopsis* protoplasts (Fig. 4), we postulate that GsCLC-c2 probably affects this by increasing the sequestration of Cl^−^ into the root cell vacuoles. Similarly, under salt stress, K^+^ contents in the stems and leaves were increased, and Na^+^ levels and Na^+^/K^+^ ratios were both reduced in the aerial parts while raised in the roots of *GsCLC-c2*-overexpressing hairy roots composite soybean plants, implying more Na^+^ has been sequestered in the roots of these transgenic plants than in the controls (Fig. 6d-f). It is largely unknown how CLC regulates the Na^+^/K^+^ ratio. Yet, it has been suggested that Cl^−^ and Na^+^ transportation is somehow coupled [14]. Alteration of one would sometimes result in the alteration of the other [27, 40]. Furthermore, whether *GsCLC-c2* is directly involved in the Cl^−^ accumulation or sequestration in the vacuoles of the roots of the transgenic composite soybean plants under salt stress, or whether it indirectly contributes to the restriction of Cl^−^ transport from roots to shoots requires further investigations.

In Arabidopsis, whether the anion channel proteins, AtCLCa-d and AtCLCg, have a higher affinity for NO_3_^−^ or Cl^−^ is determined by the amino acid “x” in the highly conserved domain I – GxGIPE. If x is P (proline), NO_3_^−^ would be the preferred anion; if x is S (serine), Cl^−^ would be preferred [24, 26]. GmCLC1, with 78% identity to AtClCa, is the first CLC reported in soybean and has a proline residue (P165) in the conserved domain I [23, 26]. Our previous study showed that GsCLC-c2 has a serine residue (S184) in the same conserved domain [23], and here we demonstrated that GmCLC1 did not show a preference between Cl^−^ and NO_3_^−^ whereas GsCLC-c2 had a slightly higher response to Cl^−^ over NO_3_^−^ (Fig. 8b).

Heterologous expression in special yeast mutants has been exploited by many researchers aiming to functionally characterize ion transporters from plant [21, 29, 30, 41, 42]. The GEF1-deficient yeast mutant (*Δgef1*)-based system was used to determine the efficacy of *GmCLC1* in its ability to alleviate chloride/salt stress or its protective functions under Cl^−^ stress [21]. In this study, the soybean *GsCLC-c2* gene was able to complement the salt-sensitive phenotype of the *△gef1* mutant grown in YPG media containing KCl or NaCl (Fig. 3a). When GsCLC-c2 was mutated by one amino acid substitution (S184P, E227V, and E294G in the conserved domains I, II, and III, respectively), its ability to complement the *gef1* mutation in yeast was very much abolished. The ability of the S184P-transformed yeast mutant to accumulate Cl^−^ in the cells was also drastically reduced (Fig. 3b), which could be the result of changing the Cl^−^ or NO_3_^−^ specificity by the presence of either serine or proline in the determining position within the conserved domain I. However, the other mutations, such as C638F, A746T and I71V/C165Y within the non-conserved areas had no effect on the ability of GsCLC-c2 to complement the restrained growth of the *△gef1* mutant in media containing KCl, NaCl (Fig. 3). The *△gef1* mutant transformed with these mutated forms of *GsCLC-c2* also accumulated comparable levels of Cl^−^ to those transformed with the wild type *GsCLC-c2* (Fig. 3b), indicating that these non-conserved domains may not play any part in the protein’s function as an anion/chloride channel. This result also implied that introducing these amino acid substitutions has little effect on the function of this protein by affecting its conformation (Fig. 3).

The *X. laevis* oocyte, a heterologous expression system, has been successfully used to study various biological processes and ion channels [30, 43]. In this work, GsCLC-c2-expressing *X. laevis* oocytes displayed a significantly elevated voltage-dependent conductance when bathed in a solution containing 96 mM NaCl, and this was negated by the inhibitory effect of the chloride channel blocker, NPPB (Fig. 7a). Furthermore, the conductance exhibited by the GsCLC-c2-expressing oocytes was elevated in proportion to increasing Cl^−^ concentrations (Fig. 7b, Additional file 1: Figure S1), but, surprisingly, independent of pH (Fig. 7c). The latter indicates that the Cl^−^ transport function of GsCLC-c2 does not involve H^+^ exchange, and this is not consistent with the functions of AtCLCa*,* which can transport Cl^−^ or NO_3_^−^ reversely coupled with H^+^ transport, and its affinity for NO_3_^−^ is stronger than for Cl^−^ [43, 44]. GsCLC-c2 is also different from GmCLC1, whose anion transport function showed pH dependenc**e** [30]. It appears that GsCLC-c2 is an anion channel and not a H^+^/anion cotransporter as deduced from our previous bioinformatics analyses [23]. Therefore, further studies of the functions of GsCLC-c2 are required.

## Conclusions

*GsCLC-c2* is up-regulated in the roots of NaCl-stressed wild soybean plants, and the encoded protein is more effective than GmCLC1 as a chloride channel, with higher permeability to Cl^−^ and higher affinity for Cl^−^ than NO_3_^−^ than GmCLC1, and is responsive to halide ions in general. Its chloride transport function is pH-independent, but dependent on the amino acid sequences in the conserved domains I, II and III. It is localized in the tonoplast and therefore likely contributes to enhanced Cl^−^/salt tolerance in plants by increasing the sequestration of excess Cl^−^ in the vacuoles of root cells and thus preventing Cl^−^ from being transported to the shoots where it can result in cellular damages.

## Methods

### Plant materials, bacteria and yeast strains, *Xenopus laevis* oocytes, and plasmids

Plant seeds, including *Glycine max* (L.) Merr. cultivar N23674 (salt-sensitive) were obtained from National Center for Soybean Improvement, Key Laboratory of Biology and Genetic Improvement of Soybean, Nanjing Agricultural University, Nanjing, China. Seeds of *G. soja* accession BB52 (salt-tolerant) were collected from the coastal area in Shandong province of P.R. China [8, 12]. No special permissions were required to use this wild soybean accession. Wild-type (WT) *Arabidopsis thaliana* Columbia-0 *glabrous1–1* (Col-0 *gl1–1*, alias Col-5) was a gift from Dr. H.Z. Shi lab of Texas Tech University, USA. *Escherichia coli* DH5α, *Agrobacterium rhizogenes* strain K599, plant transient expression vectors pJIT166-GFP and pJIT166-RFP, binary vector for plant transformation pCAMBIA1300, yeast mutant *Δgef1* in BY4741 background and yeast expression plasmid pYES2, *Xenopus laevis* oocytes and plasmid pGEHME for the generation of cRNA were used in this study.

### Cloning of soybean *CLC* genes and vector construction for plant, bacterial and yeast transformation

The seed germination and seedling cultivation of soybean were conducted as previously reported [21]. Total RNA was extracted from 15-day-old soybean seedlings using the TaKaRa MiniBEST Plant RNA Extraction Kit (TaKaRa, Dalian, China). The full-length coding sequence (CDS) of *CLC* genes were amplified in PCR reactions containing 1× PCR buffer, 0.15 mM MgCl_2_, 0.25 mM dNTPs, 0.2 μM of each primer, 0.25 U KOD-Plus DNA polymerase (TOYOBO, Japan), and 2 μL first-strand cDNA produced with PrimeScript™ II 1st Strand cDNA Synthesis Kit (TaKaRa, Dalian, China) according to the manufacturer’s protocol. The PCR products were ligated to the pMD 19-T vector (TaKaRa, Dalian, China) and sequencing of the clones was done using the sequencing primer sites on the vector. Primer sequences are listed in Additional file 1: Table S2.

The CDS of *GsCLC-c2* without the stop codon was cloned upstream of and in-frame with the CDS of green fluorescent protein (GFP) in the pJIT166-GFP plasmid to obtain the recombinant plasmid pJIT166-*GsCLC-c2-GFP*. The tonoplast marker gene, *δ-TIP*, for co-localization was cloned in-frame with the 3′-terminus of red fluorescent protein (RFP) CDS in the pJIT166-RFP plasmid to obtain pJIT166-*RFP-δ-TIP* for Arabidopsis leaf protoplast transformation [39, 45]. The CDS of *GsCLC-c2* was cloned into pCAMBIA1300 to obtain the pCAMBIA1300-*GsCLC-c2*, which was then transformed into *A. rhizogenes* K599 for plant transformation. To express *GsCLC-c2* in the yeast mutant *Δgef1*, the CDS was cloned into the yeast expression vector pYES2. To express *GsCLC-c2* and *GmCLC1* in *X. laevis* oocytes, their CDS were cloned into pGEHME. Primers used for amplifying the above genes are shown in Additional file 1: Table S2. pCAMBIA1300-*GmCLC1* was constructed as our previous work [21].

### Chromosomal location determination

In order to pinpoint the distribution of *GmCLCs* family members throughout the soybean genome, we used each member as a query against the soybean genome on the SoyBase Wm82 Genome Browser (https://www.soybase.org/gb2/gbrowse/gmax2.0/) to determine the location of the transcription initiation site of each gene. The MapInspect software (version 1.0) was used to draw the locations of *GmCLC* genes on each chromosome.

### qRT-PCR assays

The roots, stems, and leaves were sampled from 15-day-old N23674 and BB52 plants treated with 150 mM NaCl for 0, 2, 4, 8, 12, and 24 h, respectively, and were frozen in liquid N_2_ for RNA extraction. Total RNAs were isolated using MiniBEST Plant RNA Extraction Kit (TaKaRa, Dalian, China) and then used for the 1st strand cDNA synthesis following the instructions of PrimeScript RT reagent Kit with gDNA Eraser (TaKaRa). A soybean elongation factor 1-alpha-like gene, *EF1α2a* (XM_003524541.1) [46], was used as an internal reference. qRT-PCR reactions were performed in 96-well plates using the StepOnePlus Real-Time PCR System (ThermoFisher Scientific China, Inc., Shanghai) with SYBR® Premix Ex Taq™ II (TaKaRa) according to manufacturer’s protocols in a 10-μL reaction. Data were analyzed by StepOnePlus Software v2.1 (Thermo Fisher Scientific China, Inc., Shanghai). The relative expression of each gene was normalized against the internal reference gene, and calculated according to the 2^−ΔΔCT^ method [47]. Primers used in the qRT-PCR are presented in Additional file 1: Table S2.

### Subcellular co-localization assays of GsCLC-c2

The seed germination and seedling cultivation of Arabidopsis were conducted as previously reported [21]. Arabidopsis protoplasts were isolated from the leaves of 25-day-old plants as previously reported [48]. Two fusion constructs *GsCLC-c2-GFP* and tonoplast specific marker *RFP-****δ****-TIP* [39] were co-transformed into the protoplast by the PEG4000-mediated method [49]. After incubation of the transformed Arabidopsis protoplasts for 18–24 h at room temperature, GFP and RFP signals were detected by confocal fluorescence microscopy (PerkinElmer UltraVIEW VoX).

### Site-directed mutagenesis (SDM)-mediated complementation tests of *GsCLC-c2* in yeast mutant

To investigate the Cl^−^ transport functions of GsCLC-c2, site-directed mutagenesis of *GsCLC-c2* was carried out. The strategies for the SDM of *GsCLC-c2* are shown in Additional file 1: Table S3, and the mutations were performed with the Muta-direct™ site-directed mutagenesis Kit (SBS Genetech, Shanghai, China) using the pYES2-*GsCLC-c2* construct according to manufacturer’s protocol. The desired constructs confirmed with sequencing were transformed into the salt-sensitive yeast mutant *△gef1* as described previously [50]. The yeast cultures in 10-fold dilution series beginning at OD_600_ ≈ 0.5 were plated on YPD medium (1% yeast extract/2% peptone/2% dextrose), YPG medium (1% yeast extract/2% peptone/2% galactose), with or without 1 M NaCl, or 1 M KCl supplement. The plates were photographed 2~4 days after incubation at 30 °C. Cl^−^ contents of WT (*S. cerevisiae* BY4741), *Δgef1* mutant, *Δgef1*/*GsCLC-c2*, or *Δgef1/GsCLC-c2* mutants were determined with cultures grown in liquid YPG medium with 1 M NaCl. Cells were harvested at OD600 ≈ 0.2 by filtration, and their Cl^−^ content was determined [51].

### Salt tolerance assays of *GsCLC-c2* overexpressing hairy root composite soybean plants

Hairy root transformation was performed as described in our previous work [21]. Soybean seedlings were transformed with pCAMBIA1300 (Vector), pCAMBIA1300-*GsCLC-c2* or pCAMBIA1300-*GmCLC1* using *A. rhizogenes* strain K599. Ten-day after K599 infection, seedlings with similar length of hairy roots were transferred to ½ X Hoagland solution with or without 120 mM NaCl. Solution was replaced every 3 days. Roots, stems and leaves were harvested 7-day after transfer for Na^+^, K^+^, Cl^−^ and NO_3_^−^ content measurement [51, 52]. Photos of plants were taken right before sample harvest.

### *Xenopus laevis* oocyte transformation with *GsCLC-c2* by microinjection and electrophysiology

Ambion mMESSAGE mMACHINE™ T7 Transcription Kit (AM1344, Life Technologies, California, United States) was used to synthesize capped cRNAs from linearized pGEHME-*GsCLC-c2* and pGEHME-*GmCLC1*. Harvesting and handling of *X. laevis* oocytes were done as previously described [30]. Oocytes were injected with either 50 nL of H_2_O or 50 nL of 1 ng L^− 1^ cRNA. The injected oocytes were incubated in the standard bath solution (96 mM NaCl, 2 mM potassium gluconate, 5 mM calcium D-gluconate, 1.2 mM MgSO_4_, 5 mM HEPES, pH 7.5) at 16~18 °C [43] before two-electrode voltage clamping. Data acquisition and analysis were done using the oocyte clamp recording interface (Warner Instrument OC-725C, Hamden, CT, USA) and pClamp9 software (Molecular Devices, San Jose, CA, USA) [30]. To verify whether GsCLC-c2 is a bona fide Cl^−^ transporter, oocytes expressing the transporter were challenged with bath solution with different concentration of Cl^−^ and 10 μM NPPB (5-nitro-2-[3-phenylpropylamino] benzoic acid, an anion channel inhibitor) [31]. In order to determine the effect of extracellular pH on the transporter function in the range of 5.5 to 8.5, the bath solution was buffered with 5 mM MES for pH 5.5, and with 5 mM Tris for pH 8.5. In ion substitution experiments, 96 mM NaCl was substituted with equal concentrations of NaI, NaBr or NaNO_3_. Ag/AgCl electrodes and 3 M KCl agar bridges were used as reference and bath electrodes, respectively [30].

### Statistical analyses

All data were analyzed and presented as the means ± SD for each treatment (*n* = 3; except for electrophysiology where *n* = 4~9) using SPSS software (ver. 20.0). The data were subjected to the one-way analysis of variance (ANOVA), and pairwise comparisons were performed using Duncan’s test at *P* ≤ 0.05. For physiological data, data of different transgenic lines under control and NaCl treatment conditions were compared. For ion content, data of different transgenic lines among the same tissue under control and NaCl treatment conditions were compared.

## Additional file

Additional file 1: **Figure S1.** Pearson correlation of membrane currents of *Xenopus* oocytes injected expressing *GsCLC-c2*. **Figure S2.** Comparison of chloride transporting activity of GmCLC1 and GsCLC-c2. **Table S1.** Summary of soybean *CLC-*homologous genes information in *G. max* (cultivar N23674) and *G. soja* (accession BB52). **Table S2.** Primers for soybean *CLC* genes analyses. **Table S3.** Information for site-directed mutagenesis of *GsCLC-c2 (DOCX 140 kb)*

## Abbreviations

ANOVA: Analysis of variance
CDS: Coding sequence
CLC: Chloride channel
Col-0 *gl1–1*: *Arabidopsis thaliana* Columbia-0 *glabrous1–1*
GFP: Green fluorescent protein
NPPB: 5-nitro-2-(3-phenylpropylamino) benzoic acid
PSII: Photosystem II
REL: Relative electrolyte leakage
RFP: Red fluorescent protein
RWC: Relative water content
SDM: Site-directed mutagenesis
WT: Wild-type
YPD: Yeast extract, peptone, dextrose
YPG: Yeast extract, peptone galactose
