# Supplementary material for: Identification and functional characterization of the chloride channel gene, GsCLC-c2 from wild soybean
Source: BMC Plant Biol. 2019 Apr 1;19:121. doi: 10.1186/s12870-019-1732-z (PMC6444504; doi:10.1186/s12870-019-1732-z)
Supplement: Supplementary file 1 — Figure S1. Pearson correlation of membrane currents of Xenopus oocytes injected expressing GsCLC-c2. Figure S2. Comparison of chloride transporting activity of GmCLC1 and GsCLC-c2. Table S1. Summary of soybean CLC-homologous genes information in G. max (cultivar N23674) and G. soja (accession BB52). Table S2. Primers for soybean CLC genes analyses. Table S3. Information for site-directed mutagenesis of GsCLC-c2 (DOCX 140 kb) [file 12870_2019_1732_MOESM1_ESM.docx]

**Identification and functional characterization of the chloride channel gene, *GsCLC-c2* from wild soybean**

Running title: Characterization of *GsCLC-c2* from wild soybean

Peipei Wei^1#^, Benning Che^1#^, Like Shen^2#^, Yiqing Cui^1^, Shengyan Wu^1^, Cong Cheng^1^, Feng Liu^1^, Man-Wah Li^3^, Bingjun Yu^1,*^ and Hon-Ming Lam^3,*^

^#^ contributed equally to this work.

**Supplementary Materials**

**Figure S1.** Pearson correlation of membrane currents of *Xenopus* oocytes injected expressing *GsCLC-c2*.

**Figure S2.** Comparison of chloride transporting activity of GmCLC1 and GsCLC-c2.

**Table S1.** Summary of soybean *CLC-*homologous genes information in *G. max* (cultivar N23674) and *G. soja* (accession BB52).

**Table S2.** Primers for soybean *CLC* genes analyses.

**Table S3.** Information for site-directed mutagenesis of *GsCLC-c2*.

**
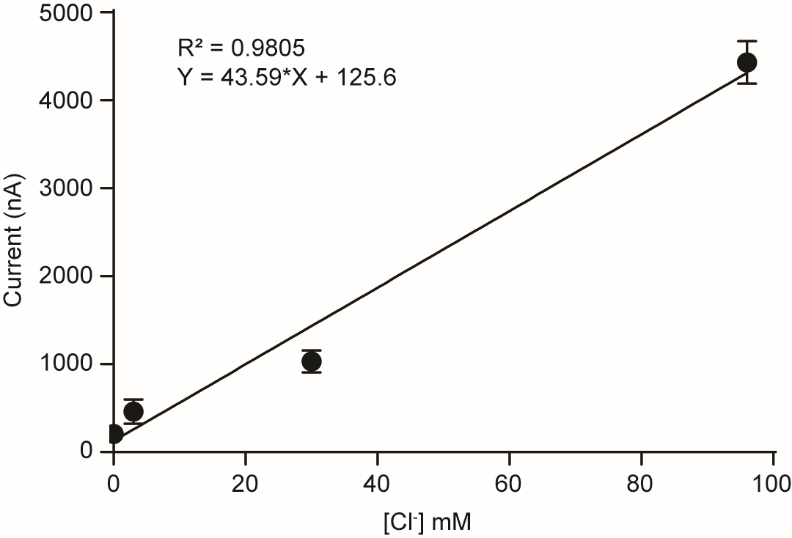
**

**Figure S1.** Pearson correlation of membrane currents of *Xenopus* oocytes expressing *GsCLC-c2*. Currents measured at 100 mV was plotted against the concentration of Cl^-^ in the bath solution. For each concentration, membrane current of at least 5 oocytes were measured (Fig. 7B). Mean ± SD.

**
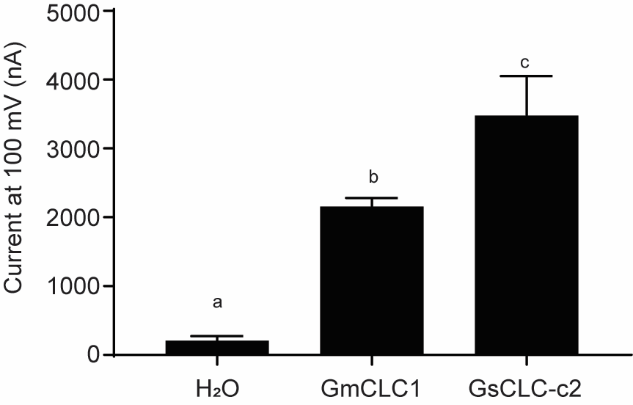
**

**Figure S2.** Comparison of chloride transporting activity of GmCLC1 and GsCLC-c2. Membrane current at 100 mV of oocytes injected with water, *GmCLC1* cRNA or *GsCLC-c2* cRNA were shown. Letters indicated groups with statistical difference. Data was analyzed with one-way ANOVA followed by Duncan’s test.

**Supplementary Table S1.** Summary of soybean *CLC-*homologous genes information in *G. max* (cultivar N23674) and *G. soja* (accession BB52).

| **Soybean *CLC* genes** | **Chromosome location** | **CDS length**  **(bp)** | **Nucleotide substitution** | **Amino acid substitution** |
| --- | --- | --- | --- | --- |
| *GmCLC1* | 5 | 2322 | — | — |
| *b1* | 16 | 2178 | 1751 (T-G)  1906 (A-G) | — |
| *b2* | 19 | 2373 | 1737 (T-C) | — |
| *c1* | 9 | 2370 | — | — |
| ***c2*** | 16 | 2391 | 183 (G-A)  **462 (T-A)**  1914 (G-T)  2181 (A-C) | —  **154 (D-E)**  —  — |
| *d1* | 1 | 2406 | — | — |
| *d2* | 11 | 2472 | 366 (C-T)  1332 (C-T)  1561 (G-A)  1771 (G-A) | —  —  —  — |
| *g* | 13 | 2298 | 834 (T-A) | — |

**Note:** “—”: no differences in nucleotide or amino acid sequences in cultivar N23674 (*G. max*) and accession BB52 (*G. soja*). The numbers in the 4^th^ and 5^th^ columns represent the substitutions of nucleotides and amino acids between N23674 and BB52, the first letter of a pair in bracket represents the nucleotide/amino acid in N23674, and the second letter represents that in BB52.

**Table S2.** Primers for soybean *CLC* genes analyses.

| **Purposes** | **Genes** | **Forward primers (5' to 3')** | **Reverse primers (5' to 3')** |
| --- | --- | --- | --- |
| Gene cloning | *GmCLC1* | ATGGGTGAGGAATCCAGTTT | TCACTTCCTCTTTGATTTTGC |
|  | *b1* | ATGGGAGAGGATTCTAGGGAGT | CTAGCACTGTAAAGGGAGTGTATTG |
|  | *b2* | ATGGGAGAGGATTCTGGGGA | TCAATTCCTCTTTTCTCTACCCTTA |
|  | *c1* | ATGATGACACCAACCGGC | TCATTTCCATTTGTGAGGCAT |
|  | *c2* | ATGACACCAACCAGCGGCGAA | TCATTGCCATTTGTGAGGCTTGAT |
|  | *d1* | ATGCTGTCCAATCATTTCCAA | TTAGTCAGGTATCTGATTCTGAAG |
|  | *d2* | ATGCTGGCCAATCATTTCC | TTAGTCAGGTATCTGATTTTGAAGC |
|  | *g* | ATGTCCACGAACTACTCAACCAA | TTAAAATCTTAAACTTTTCCCTGTGT |
| qPCR | *GmEF1α2a* | GGATGTCGTTTCTTATGGT | CAAACACAACACATTAAAACAG |
|  | *GmCLC1-F* | TGGCTGAGAGAGAAGGGAATA | GTGAAGGGTGTAGTATTGGTGAG |
|  | *b1* | GGCTGAGAGAGAAGGAAGTATTG | CTAGCACTGTAAAGGGAGTGTATT |
|  | *b2* | GCATCTACATGGGACCTCATAC | AGGAAGATCACACAAAGGGATAC |
|  | *c1* | GGTTCCATCCTAGGAGTTTCTG | ATCCACCCTGACCCAATAAAG |
|  | *c2* | CCACCTCCCAGATTGCTATTATT | CCTGGAACTCCAGTCATTCTTC |
|  | *d1* | GTGTCGAGAGCCTTGACTATG | CCATTTCACCACCAGCAAATAG |
|  | *d2* | GGGAGCAGGGTCAAGATTATT | CAGAATCAGGGCAAGGACTAC |
|  | *g* | GCATCTACATGGGACCTCATAC | AGGAAGATCACACAAAGGGATAC |
| Subcellular co-localization | *GsCLC-c2* | GCGTCGACATGACACCAACCAGC | CGGGATCCTTGCCATTTGTGAGGC |
|  | *δ-TIP* | GACGCTGGAGTTGCCTTTGGTTC | TTAGAAATCAGCAGAAGCAAGAGG |
| Plant expression | *GmCLC1* | GGGGTACCATGGGTGAGGAATCCAGTTT | GGACTAGTCTTCCTCTTTGATTTTGCCAG |
|  | *GsCLC-c2* | TCTAGAATGACACCAACCAGCG | GGATCCTTGCCATTTGTGAGGC |
| Yeast Complementation | *GsCLC-c2* | CGGGATCCATGACACCAACCAG | CCGCTCGAGTCATTGCCATTTGTGA |
|  | *GEF1* | CGGGATCCATGCCAACAACTTATG | CCGCTCGAGTCATATAACGTTACCAT |
| Electro-  physiology | *GmCLC1* | CGGAATTCATGGGTGAGGAATCC | GCTCTAGATCACTTCCTCTTTGATTT |
|  | *GsCLC-c2* | CGGGATCCATGACACCAACCAG | GCTCTAGATCATTGCCATTTGTGAG |

**Table S3.** Information for site-directed mutagenesis of *GsCLC-c2*.

| **Nucleotide**  **position** | **Amino acid position** | **Conserved domains** | **Predicted amino acid** | **Mutated**  **amino acid** | **Forward primers (5' to 3')** | **Reverse primers (5' to 3')** |  |
| --- | --- | --- | --- | --- | --- | --- | --- |
| 550 (TCT→CCT) | 184 | Ⅰ | Ser (S) | Pro (P） | CCAGCAGCTGCAGGCCCTGGCATTCCAGAGGT | ACCTCTGGAATGCCAGGGCCTGCAGCTGCTGG |  |
| 680 (GAG→GTG) | 227 | Ⅱ | Glu (E) | Val (V) | GTGGTGGGCAAAGTGGGGCCTATGGTACAT | ATGTACCATAGGCCCCACTTTGCCCACCAC |  |
| 881 (GAG→GGG) | 294 | Ⅲ | Glu (E) | Gly (G) | CTTTGCTCTTGAAGGGGCAGCTACATGGTG | CACCATGTAGCTGCCCCTTCAAGAGCAAAG |  |
| 1914 (TGC→TTC) | 638 | - | Cys (C) | Phe (F) | GATCTCTTGTTACTTCCACCTGTAATGTTGGTC | GACCAACATTACAGGTGGAAGTAACAAGAGATC |  |
| 2236 (GCT→ACT) | 746 | - | Ala (A) | Thr (T) | CACTTGCCAAAGCTACTATTCTTTTCCGCC | GGCGGAAAAGAATAGTAGCTTTGGCAAGTG |  |
| 211 (ATT→GTT) /  495 (TGT→TAT) | 71/ | - | Ile (I) / | Val (V) / | CCAGATTGCTATTGTTGGTGCCAACCTCTGCCC | GGGCAGAGGTTGGCACCAACAATAGCAATCTGG |  |
|  | 165 |  | Cys (C) | Tyr (Y) | GCTGGTGCGAATATGTATTTAGCAGCAGCTGC | GCAGCAGCTGCTGCTAAATACATATTCGCACCA | |
